# Investigating the impacts of intrafraction motion on dosimetric outcomes when treating small targets with virtual cones

**DOI:** 10.1002/acm2.13285

**Published:** 2021-07-17

**Authors:** Cody Church, David Parsons, Alasdair Syme

**Affiliations:** ^1^ Department of Physics and Atmospheric Science Dalhousie University Halifax NS Canada; ^2^ Department of Radiation Oncology University of Texas Southwestern Medical Center Dallas TX USA; ^3^ Department of Radiation Oncology Department of Physics and Atmospheric Science Dalhousie University Halifax NS Canada

**Keywords:** Monte Carlo, stereotactic radiosurgery, virtual cones

## Abstract

**Purpose:**

Intrafraction patient motion is a well‐documented phenomenon in radiation therapy. In stereotactic radiosurgery applications in which target sizes can be very small and dose gradients very steep, patient motion can significantly impact the magnitude and positional accuracy of the delivered dose. This work investigates the impact of intrafraction motion on dose metrics for small targets when treated with a virtual cone.

**Materials and Methods:**

Monte Carlo simulations were performed to calculate dose kernels for treatment apertures ranging from 1 × 2.5 mm^2^ to 10 × 10 mm^2^. The phantom was an 8.2‐cm diameter sphere and isotropic voxels had lengths of 0.25 mm. Simulated treatments consisted of 3 arcs: 1 axial arc (360° gantry rotation, couch angle 0°) and 2 oblique arcs (180° gantry rotation, couch angle ±45°). Dose distributions were calculated via superposition of the rotated kernels. Two different collimator orientations were considered to create a virtual cone: (a) each treatment arc was delivered twice, once each with a static collimator angle of ±45°, and (b) each treatment arc was delivered once, with dynamic collimator rotation throughout the arc. Two different intrafraction motion patterns were considered: (a) constant linear motion and (b) sudden, persistent motion. The impact of motion on dose distributions for target sizes ranging from 1 to 10 mm diameter spheres was quantified as a function of the aperture size used to treat the lesions.

**Results:**

The impact of motion on both the target and the surrounding tissue was a function of both aperture shape and target size. When a 0.5‐mm linear drift along each dimension occurred during treatment, targets ≥5 mm saw less than a 10% decrease in coverage by the prescription dose. Smaller apertures accrued larger penalties with respect to dosimetric hotspots seen in the tissues surrounding the target volume during intrafraction motion. For example, treating a 4‐mm‐sized target that undergoes 2.60 mm (3D vector) of continuous linear motion, the D_5_ in the concentric shells that extend 1, 2, and 3 mm from the surface of the target was 39%, 24%, and 14% smaller, respectively when comparing the delivery of a larger aperture (6 × 10 mm^2^) to a smaller aperture (2 × 5 mm^2^). Using a static collimator for shaping a virtual cone during treatment minimized the dosimetric impact of motion in the majority of cases. For example, the volume that is covered by 70% or more of the prescription dose is smaller in 60.4% of cases when using the static collimator. The volume covered by 50, and 30% or more of the prescription dose is also smaller when treating with a static collimator, but the clinical significance of this finding is unknown.

**Conclusions:**

In this work, the dosimetric trade‐offs between aperture size and target size when irradiating with virtual cones has been demonstrated. These findings provide information about the tradeoffs between target coverage and normal tissue sparing that may help inform clinical decision making when treating smaller targets with virtual cones.

## 1 Introduction

Highly conformal treatments of small cranial lesions utilize a technique known as Stereotactic Radiosurgery (SRS) which aims to achieve sub‐mm target localization in all three spatial dimensions.^1^ Compared with conventionally‐fractionated treatments, single‐fraction SRS and few‐fraction stereotactic radiotherapy (SRT) are characterized by large doses per fraction, high dose conformity, and strict patient positioning tolerances.^2^ Several approaches have been developed to deliver these treatments, including VMAT and stereotactic cones. In comparison to VMAT, cones have demonstrated better conformity for smaller target volumes (4 mm in diameter) when treating spherical lesions.^3^, ^4^ For certain indications such as trigeminal neuralgia (TN) where targets sizes become sufficiently small and dose limitations on surrounding tissues are stringent,^5^ circular stereotactic cones are most commonly used for treatment delivery.^6^

Recent literature has demonstrated that a combination of collimator rotations and apertures shaped by the multi‐leaf collimator (MLC), referred to as a virtual cone, are capable of shaping dose distributions comparable to stereotactic cones for small targets. Popple *et al* aimed to create spherical dose distributions for the purpose of treating a small target like the trigeminal nerve with a virtual cone and found that performing an arc‐based delivery with a 2.1 × 5 mm^2^ aperture using two arcs with orthogonal collimator angles, produced a dose distribution comparable to a 4‐mm stereotactic cone defined at the 50% isodose line.^7^ Additional preliminary work with virtual cones investigated the treatment of functional disorders (e.g., thalamotomy of the nucleus ventralis intermedius (VIM)), which coupled high‐resolution fMRI and SRS to delineate and ablate the VIM.^8^ They found that a delivery with a fixed‐MLC position and series of non‐coplanar arcs can deliver a spherical dose distribution comparable to a 4‐mm SRS shot with a cone. Another study using virtual cones for dorsal nerve root ganglion ablation therapy alluded to the potential of reducing treatment times (and therefore intrafraction motion) when using virtual cones, but did not quantify the dosimetric impact of intrafraction motion with virtual cones.^9^ They found that the shape of the 60 Gy isodose surface was appropriate for the ablative doses used in therapy, and that the dose limits on surrounding organs at risk were satisfied. Furthermore, the conformity of the spherical dose profile shaped by virtual cones and arc arrangement eliminated the need for inverse planning and could be used as a standard template for most patients.

Historically, framed‐based systems were used for immobilization during SRS treatments,^10^, ^11^, ^12^, ^13^ but many centers have moved away from invasive immobilization techniques in favor of non‐invasive, thermoplastic mask‐based methods. However, mask‐based systems have been shown to allow larger intrafractional positioning errors that increase in magnitude with increasing treatment time.^14^, ^15^, ^16^ There have been several studies that have investigated the magnitude of detected motion within different thermoplastic mask systems and imaging modalities. Using BrainLAB frameless masks and imaging with the Brainlab ExacTrac stereoscopic X‐ray system, Gaevart *et al* reported the 3D displacement from intrafraction motion to be 0.66–3.16 mm.^17^ Similarly, Bichay *et al*. found 3D displacements of 0.4–3.23 mm using a Civco mask, and aligning orthogonal images to digitally reconstructed radiographs (DRR).^18^ Tryggestad et al. showed that set‐up errors could range from 2.1 to 2.7 mm with four different thermoplastic masks.^19^ Using Gamma Knife‐specific thermoplastic masks, and imaging an infrared motion marker on the nose, MacDonald *et al*. found 3D‐errors owed to intrafraction motion up to 2.5 mm.^20^ While the literature reports that the majority of patients experience sub‐mm motions, it is important to remember that pre‐treatment imaging modalities for SRS typically have 1 mm tolerances; which, in conjunction with patient motion, could lead to larger errors (>1 mm).

The dosimetric impact of motion is highly dependent upon the type of motion experienced during treatment, the magnitude of motion, and the treatment site. Previous literature has assessed the dosimetric impact of intrafraction motion on target volume (TV) coverage when treating vertebral columns with stereotactic body radiation therapy (SBRT). When treating with intensity‐modulated radiation therapy (IMRT), Kim *et al*. found that there was a ±1% median change in dose received to 95% (D_95_) and 90% (D_90_) of the target volume, the maximum dose (D_max_), and mean dose (D_mean_) for 8/9 subjects; whereas the dose received to 0.1% (D_0.1_), 0.5%(D_0.5_), 1%(D_1.0_), and D_max_ for the surrounding organs at risk (OAR) differed by −14% to 38%.^21^ Similar impacts of motion (simulated by shifts in one dimension at a time) were shown by Wang *et al*. with IMRT where a ±2 mm shift in a given dimension resulted in a reduction of up to 17.9% to the volume receiving 95% of the prescription dose (V_95_), though the majority of cases had changes of ≤5%; D_max_ to surrounding OAR differed by approximately (−15)–(+50)%.^22^ Using volumetric arc therapy (VMAT) Ong *et al*. found that a 2‐mm shift for 30s during therapy could result in a 13% increase of the maximum dose (D_max_) to the spinal cord. For cranial indications, and for TN in particular, the PTV volumes can be an order of magnitude smaller with much more stringent tolerances on positioning due to the TV abutting sensitive structures. For example, the prescription volume for TN can range from 0.001–0.05 cc. (effective spherical radius: 0.6–2.3 mm)^23^, and can reside an average of 2 mm away from the pons which is a radiologically sensitive structure.^24^ Therapeutic situations such as these necessitate planning target volume (PTV) margins to be as small as possible. However, Guckenberg showed that using a 0‐mm PTV margin on cranial lesions could result in a 40% reduction in the conformity index when intrafraction motion occurs.^25^There have been several bodies of work to investigate dosimetric impact of motion when treating larger targets (>0.52 cc) with MLC‐based VMAT,^22^, ^26^, ^27^ but there remains a gap in the literature for investigating the dosimetric impact of treating with virtual cones. This study aims to investigate the impact of intrafraction motion when treating small cranial targets with a virtual cone. Various motion traces were investigated for increasing degrees of linear drift, and sudden large motions. None of the previously published studies have investigated the dosimetric consequences of motion in a virtual cone‐based treatment delivery and previous studies related to virtual cones have restricted their analysis to a limited number of treatment apertures. Results of this study provide insight into the robustness of both target dose metrics and surrounding tissue doses when the planning conditions (no motion) differ from the treatment delivery conditions (motion) as a function of target size and treatment aperture size. Such information will be of value to clinicians seeking to understand the risk‐reward balance of highly conformal treatment apertures.

## 2 Materials and Methods

### 2.1 Monte Carlo Simulation

Dose kernels were created with the EGSnrc Monte Carlo system.^28^ To simulate a dose kernel, a phase‐space from the treatment head of the TrueBeam STx platform for a 6 MVFFF beam was provided by Varian Medical Systems through 54 phase space files (~69 Gb) that was validated down to a field size of 1 x 1 cm^2^.^29^ The phase space was scored above the jaws at 73.3 cm from isocenter, and was used an input for SOURCE‐21 containing a linac model with the jaws, HDMLC, and Mylar exit window within BEAMnrc.^30^ The MLC‐defined aperture was incident on an 8.2‐cm diameter water sphere phantom in DOSXYZnrc,^31^ with material composition defined by ICRU 521 pegs4 data file, with a 0.25‐mm isotropic voxel size. To keep voxel dose uncertainty <5% within the size of the aperture defined at the nominal isocenter (100 cm SAD), 10^8^ histories were used. Prior to applying the simulated dose kernels to dose‐delivery calculations, a Gaussian filter with a sigma of 1.2 was applied to smooth out the dose kernel. A total of 19 dose kernels were created from different apertures and are mentioned in section 2.C, and Table 1.

**Table 1:** MLC‐shaped treatment apertures.

Number of MLC leaf pairs used	Gap between leaves (mm)	Effective area (cm^2^)
1	1	0.025
	2	0.050
	3	0.075
2	1	0.050
	2	0.100
	3	0.150
	4	0.200
	5	0.250
3	2	0.150
	3	0.225
	4	0.300
	5	0.375
	6	0.450
	7	0.525
4	2	0.200
	4	0.400
	6	0.600
	8	0.800
	10	1.0000

Monte Carlo simulations of a full treatment delivery with simulated intrafraction motion (section 2.B and 2.D) were conducted with two apertures sizes (2 x 5 mm^2^, and 4 x 10 mm^2^) incident on an 8.2 cm water sphere with a 0.5 mm resolution. The simulations were conducted with a target residing in the center of the sphere, as well as targets residing 2, and 3 cm off‐axis. These simulations were then compared with the superposition methodology described in Section II.2 to quantify the impact of non‐central target locations.

### 2.2 Simulating treatment delivery

Treatments modeled in this study consisted of a set of 3 arcs: a 360° axial arc (couch angle = 0°) and 2 partial arcs (180° rotations) with the couch at ±45°. Dose distributions were calculated via superposition of the Monte Carlo‐derived dose kernels described previously. To simulate the delivery, each arc was modeled as a series of discrete control points with 10° of gantry rotation between each control point.

The kernel was rotated to account for the motion of the gantry, couch, and collimator. Rotations and translations were implemented in MATLAB utilizing tricubic interpolation.

### 2.3 Aperture size and orientation

In total, 19 different apertures shaped by a model of the NDS120HD MLC (Varian Medical Systems Inc., Palo Alto, CA) were analyzed. Although stereotactic cones can reach diameters of several cm (e.g., BrainLAB offers stereotactic cones ranging from 4 to 30 mm),^32^ this study focuses on creating dose distributions that would be comparable to plans created by stereotactic cones <10 mm in diameter. The geometric properties of the apertures studied in this work are listed in Table 1. A virtual cone was created by implementing two different arc deliveries: (a) Static Collimator: For each arc geometry in the treatment listed in section II.2, the arc was delivered twice; once each with the collimator at ±45°. (b) Dynamic Collimator: For each arc geometry, the arc was delivered once with the collimator rotating 180^o^ throughout delivery. For the axial arc, the collimator was rotated 0°–180° for half of the arc, and 180°–0° for the rest of the arc.

### 2.4 Simulating intrafraction motion

**Fig. 1:**
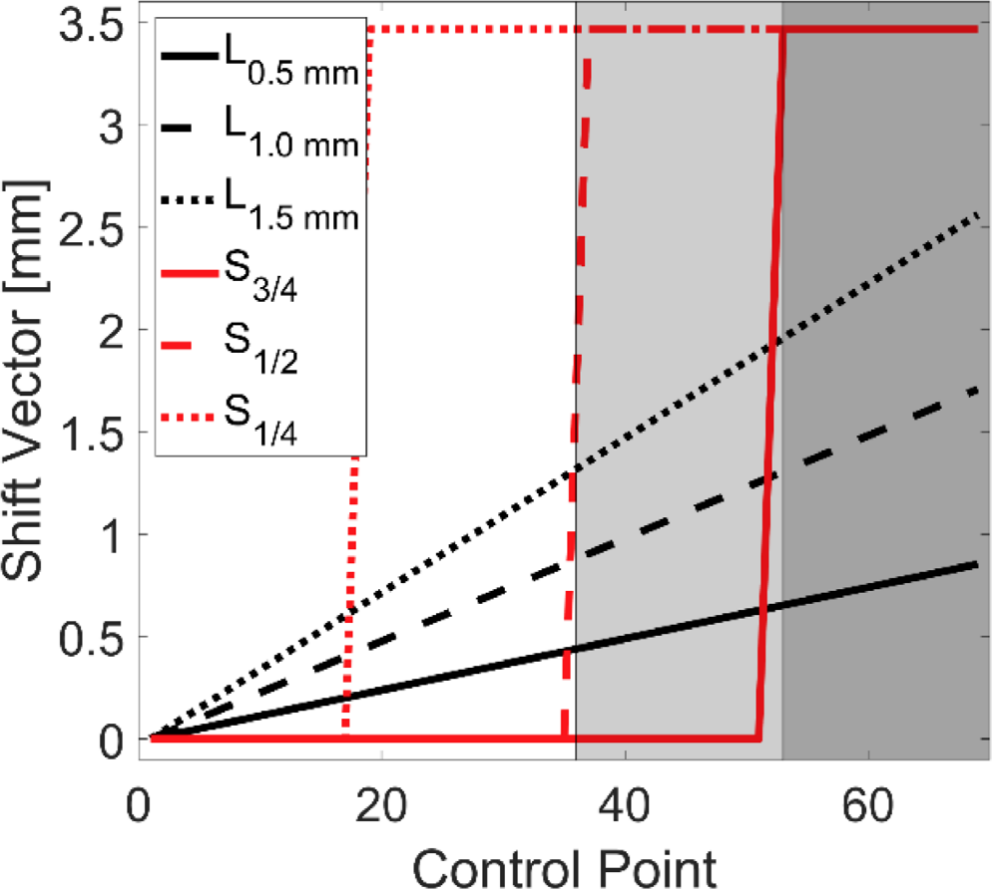
Movement traces for different intrafraction motion patterns. *L_0.5 mm_*_,_ *L_1.0 mm_*_,_ *L_1.5 mm_*_,_ represent linear motion up to 0.5, 1.0, and 1.5 mm in each dimension respectively. *S_1/4_*_,_ *S_1/2,_ S_3/4_*_,_ represents a linear motion of 2 mm in each dimension at ¼, ½, and ¾ of the way through treatment respectively. The shaded regions represent the first co‐planar arc and the two non‐coplanar arcs in order from left to right.

To approximate positioning errors owed to intrafraction motion, six different motion traces were simulated as shown in Fig. 1. Three of the traces mimicked a continuous linear drift until the phantom was offset by 0.5, 1.0, and 1.5 mm in all three dimensions which resulted in a 3D‐offset of 0.87, 1.73, and 2.60 mm, respectively (defined as **L_0.5 mm,_ L_1.0 mm,_ L_1.5 mm,_** respectively). The other three traces emulated a sudden large shift of 2 mm along each dimension (3D‐offset of 3.46 mm) at different time points during treatment, and persisted throughout treatment. These time points were chosen to occur at: ¼, ½, and ¾ of the way throughout treatment and were defined as **S_1/4__._ S_1/2,_** and **S_3/4__,_** respectively.

The spherical nature of the simulated phantom and the central location of the target meant that the dose kernel was spatially invariant. When implementing shifts of the dose kernel (caused by simulated target motion), spatial invariance was lost. To account for this, an approximation was used by calculating the intersection of the central ray for a given beam with the water sphere and applying an inverse square weighting correction based upon the magnitude of the proximal or distal shift of the ray along the beamline (assuming the entire field receives a homogenous correction).

### 2.5 Dosimetric analysis

For treatment simulations that did not involve motion, for each aperture size, a dose volume histogram (DVH) was calculated for target sizes ranging from 1 to 10 mm in diameter. For each target volume (TV), the dose matrix was normalized such that 99% of that target volume was covered by the prescription dose (which will be defined as D_99_). For simulations where motion was present, the distributions were not renormalized to achieve the same coverage. The effective output of the linac at each control point was preserved (i.e., the equivalent of delivering the same number of MU for both the no‐motion and motion cases) to facilitate evaluation of the impact of motion on delivered dose. To evaluate the dose received by the volume abutting the target, three concentric spherical shells, each with a 1‐mm thickness were created around the TV.

To evaluate the dosimetric impact of motion, the ratio of the Paddick conformity indices was calculated for the case of motion to the case without motion^33^:

$$R_{C}=\frac{TV_{M}^{2}}{PIV_{M}}\;/\;\frac{TV_{\mathrm{NO}}^{2}}{PIV_{\mathrm{NO}}}$$

where TV_*M*_ refers to the volume within the target covered by the prescription dose for the case of motion, PIV_*M*_ is the prescription isodose volume for the case of motion; both of these parameters are determined using the prescription isodose in the case of no motion. TV_*NO*_ refers to the volume within the target covered by the prescription dose for the case of no motion, PIV_*NO*_ is the prescription isodose volume for the case of no motion. A value of unity would indicate that the conformity index for the case of motion is equivalent to the case without motion.

To evaluate the steepness of the dose gradient for different plans, the gradient index (GI) was calculated by conventional means^34^:

$$GI=\frac{V_{50}}{V_{100}}$$

where V_50_ is the volume receiving 50% of the prescription dose, and V_100_ is the volume receiving 100% of the prescription dose. For this analysis, the dose distributions were normalized such that the prescription dose was defined as 100%.

## 3 Results

### 3.1 Effect of aperture size on target coverage

**Fig. 2:**
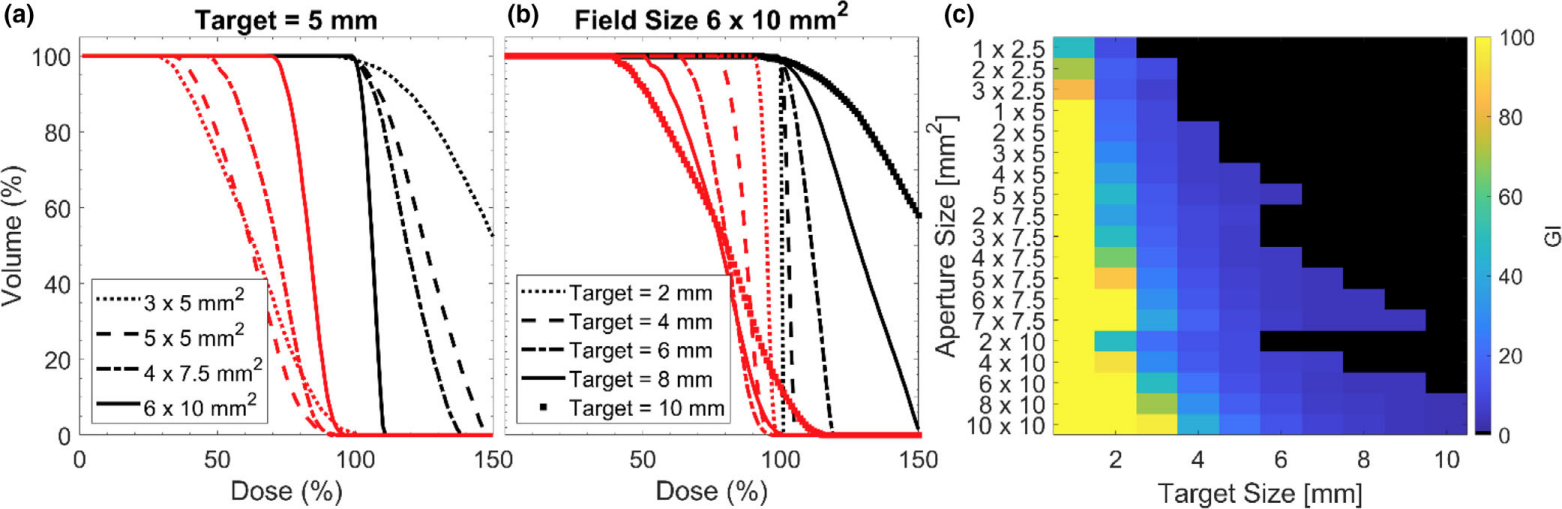
(a) Dose volume histogram for a fixed target size with various aperture sizes. Black lines represent dose to the target, red lines represent dose to the 3rd shell around the target. (b) Dose volume histogram for a fixed aperture size with various target sizes. Black lines represent dose to the target, red lines represent dose to the 3rd shell. (c) GI calculated for all field sizes and target sizes, black tiles represent a case where the maximum dose within the target was ≥ 200%.

The impact of different sized apertures on target coverage is demonstrated by the black lines in Fig. 2 for the static collimator case. In (A) a single target size (5 mm) is considered while changing the size of the aperture. For all other target sizes not shown in Fig. 2(a), the same trend of larger aperture sizes producing steeper dose volume histograms within the TV, as well as in the surrounding concentric shells is seen. Analogous data are shown in Fig. 2(b), where a fixed field size of 6 x 10 mm^2^ is used to treat various target sizes. For illustrative purposes, the doses received by the third concentric shell around the target have been included in the figures. The GI as a function of target size and aperture size is shown in Fig. 2(c). where all target size and aperture size pairings that result in a D_max_ ≥ 200% have been blacked‐out as they were considered unlikely choices for clinical application. For any given aperture size, delivering to a larger target size results in a reduction of the GI. In general, there is a trend of increasing GI as a function of effective aperture area. When implementing the dynamic collimator, the GI ranges from 6.3% smaller to 5.4% larger when compared with the static collimator. However, meaningful differences (≥2%) are seen in only 30.9% of target size and aperture combinations.

In Table 2 the minimum dose received by 100% of a volume (D_m_), and the minimum dose received by 5% of a volume (D_5_) are shown for a fixed target size with varying aperture sizes. Values are expressed as a percentage of the prescription dose. This table is representative of the trends seen within the data, which is that larger apertures (effective area) produce lower D_5_ at the expense of delivering a higher D_m_ to the surrounding concentric shells.

**Table 2:** Dose metrics for the treatment of a 5‐mm target with the static collimator case. Plans were normalized such that D_99_ was 100% of the prescription dose. D_m_ is the minimum dose received by 100% of the volume for the respective volume indicated. D_5_ is the minimum dose received by 5% of the volume for the respective volume indicated. Values are expressed as a percentage of the prescription dose.

Metric	Aperture size								
	1 x 2.5 mm^2^	1 x 5 mm^2^	3 x 5 mm^2^	5 x 5 mm^2^	3 x 7.5 mm^2^	5 x 7.5 mm^2^	7 x 7.5 mm^2^	2 x 10 mm^2^	6 x 10 mm^2^
D_5_, PTV	397	257	201	145	157	121	109	175	110
D_m_, 1^st^ Shell	54	59	59	69	71	82	91	72	90
D_5_, 1^st^ Shell	176	144	131	113	114	106	103	119	103
D_m_, 2^nd^ Shell	39	40	39	48	54	65	79	60	80
D_5_, 2^nd^ Shell	142	118	109	97	100	97	98	105	98
D_m_, 3^rd^ Shell	29	28	26	31	39	48	65	50	67
D_5_, 3^rd^ Shell	118	98	90	82	86	87	92	93	92

### 3.2 Effect of collimator orientation

**Fig. 3:**
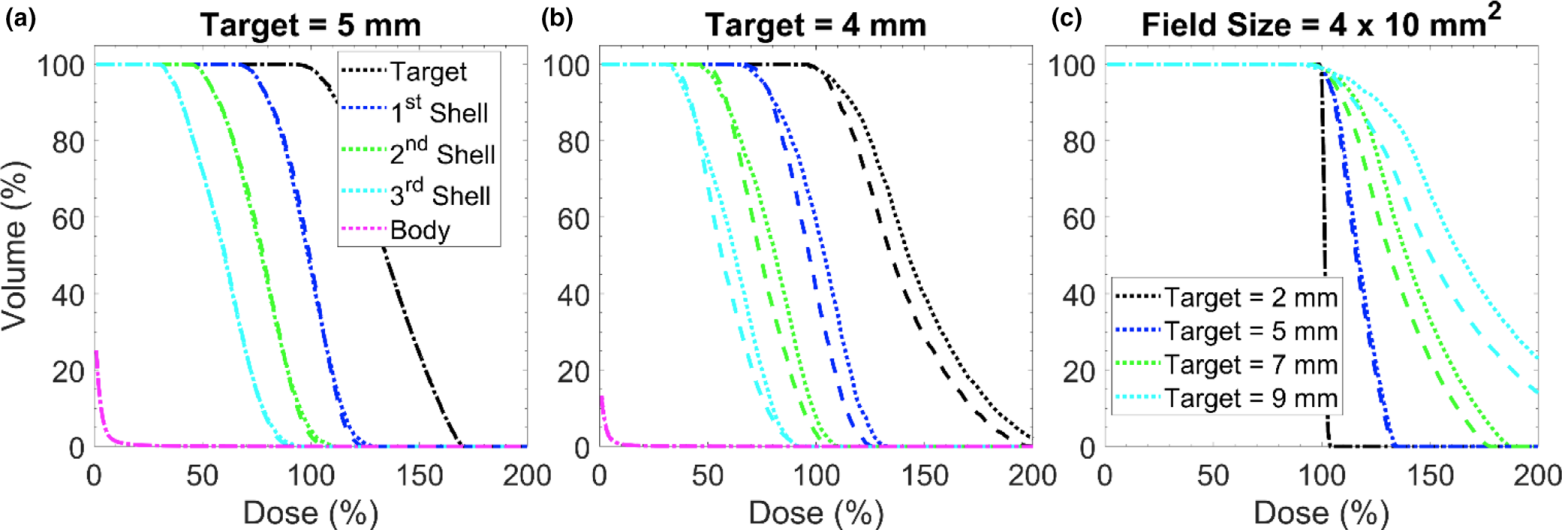
Impact of collimator orientation during delivery. (a) Dose volume histogram where target is a 5 mm sphere and aperture size is 4 x 5 mm^2^. Dotted lines represent the static collimator case, dashed lines represent the dynamic collimator case. (b) Same plot characteristics as (a) but the delivery was performed to a 4 mm target with an aperture size of 1 x 5 mm^2^. (c) A dose volume histogram for different target sizes with a fixed field size. Line definitions are the same as in (a).

The impact of collimator rotation throughout gantry motion is depicted in Fig. 3 for various circumstances. In Fig. 3.A. a collimator size of 4 x 5 mm^2^ is used to irradiate a 5‐mm spherical target. Differences between the static and dynamic collimator deliveries were minimal for both the TV and the surrounding shells. However, as shown in Fig. 3(b), when irradiating with a dynamic collimator and a 1 x 5 mm^2^ aperture, a smaller D_5_ is observed for the 1st, 2nd, and 3rd shells. The V_50_ for the dynamic collimator case is 96.7, 74.2, and 56.4%, while the D_5_ for the static collimator case is 103.9, 81.1, and 62.6% for the 1st, 2nd, and 3rd shell, respectively. The dynamic collimator creates a lower D_5_ for 82.4% of the aperture/target size combinations where the D_5_ differed by more than ±2% between the dynamic collimator and the static collimator; one of these cases is represented in Fig. 3(c) when treating a 7‐mm target. The arbitrary choice of a 2% threshold was used to highlight meaningful differences between the static and dynamic collimator deliveries as much of the data exhibited much smaller differences.

**Fig. 4:**
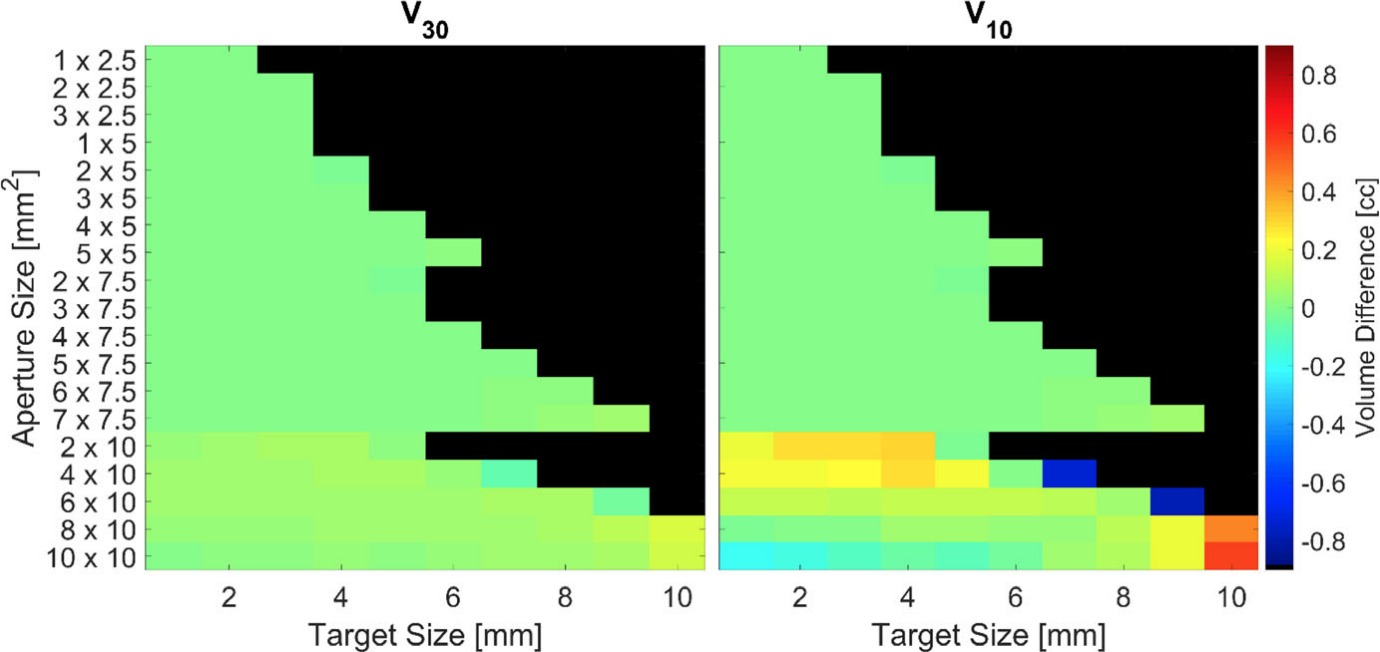
Absolute volumetric differences between the volumes receiving 30 and 10% or more of the prescription isodose defined as V_30_ and V_10_ respectively for the different collimator deliveries. Volumes <0 cc indicate a smaller relative volume for the dynamic collimator case. Blacked out tiles represent plans that delivered a D_max_ >200%.

Figure 4 depicts the absolute volumetric differences between the volumes receiving 30 and 10% or more of the prescription isodose defined as V_30_, V_10_ for the dynamic collimator compared to the static collimator. Blacked out tiles represent cases deemed to be clinically infeasible as they possess a D_max_ >200%. Volumes <0 cc indicate a smaller volume for the rotating collimator case. The magnitude of volumetric differences for V_70_ ranges from −4.31 x 10^‐2^ to 5.98 x 10^‐2^ cc, and −3.26 x 10^‐2^ to 7.72 x 10^‐2^ cc for V_50_ (data not shown). The majority of cases for V_30_, and V_10_ have volumetric differences <±0.1 cc (97.3 and 76.7% respectively). The dynamic collimator case produces smaller relative volumes in 60.4, 44.1, 41.4, and 50.5% of clinically feasible cases for V_70_, V_50_, V_30_, and V_10_, respectively.

### 3.3 Impact of motion on dosimetry

**Fig. 5:**
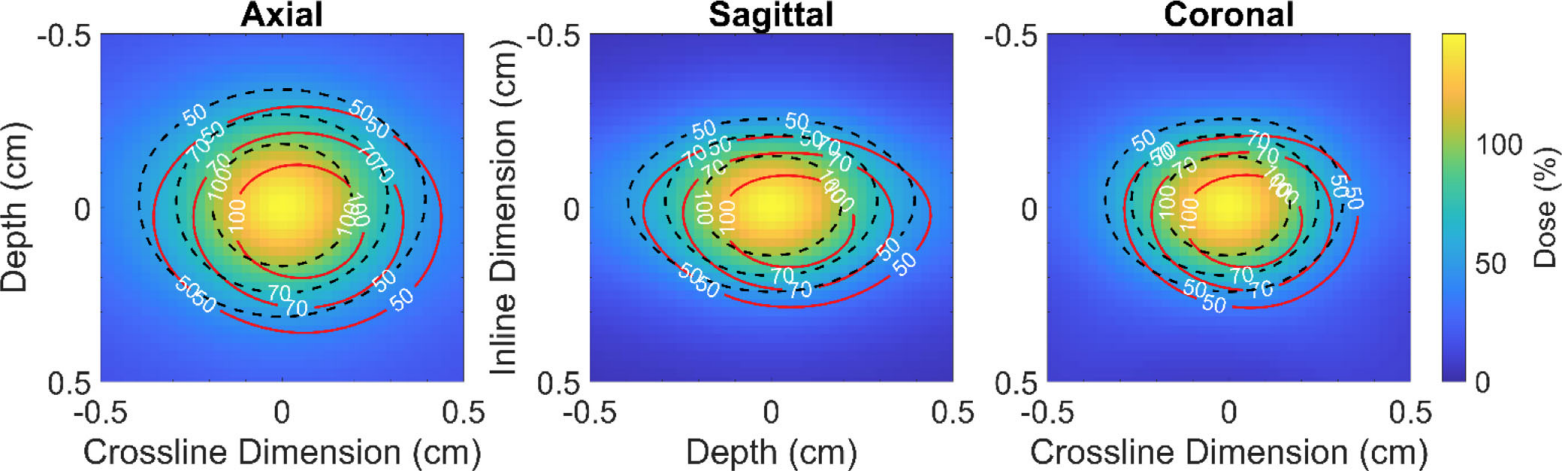
Dose map for a delivery with a 2 x 5 mm^2^ aperture with the static collimator case. The black‐dashed contour line represents delivery without motion and red lines represents with the same delivery characteristics but the phantom has been linearly moved 1.0 mm along each dimension by the end of treatment.

**Fig. 6:**
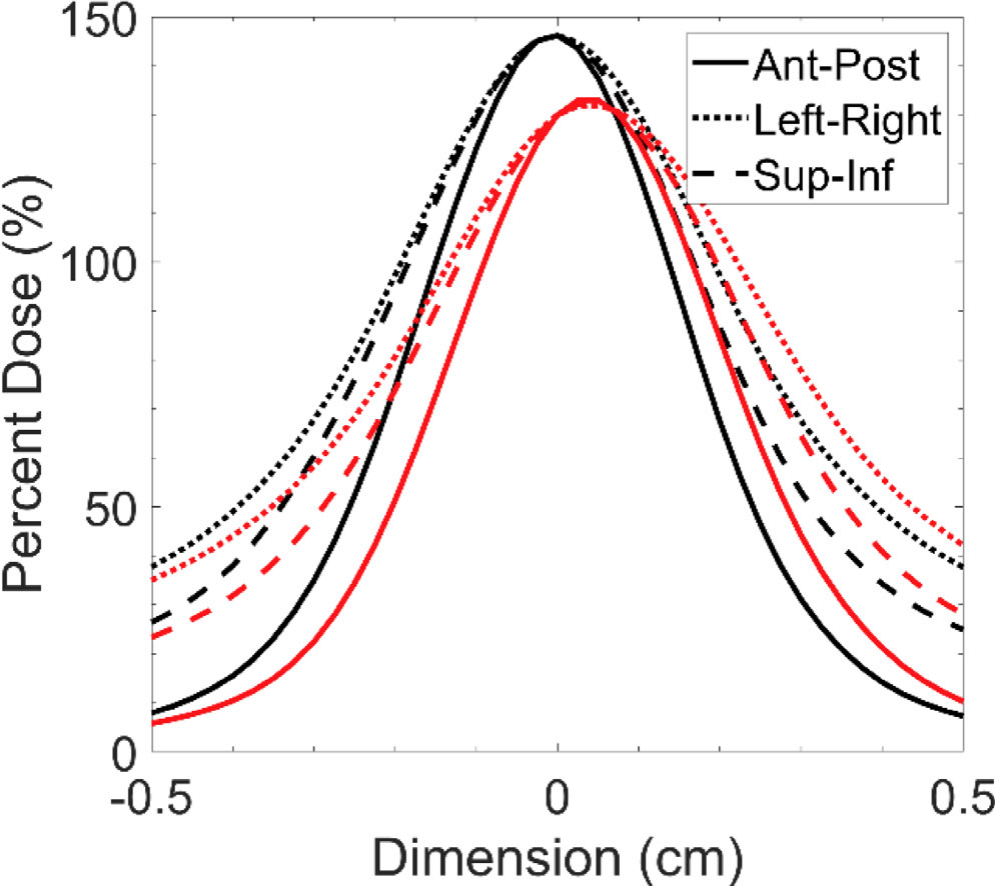
Dose profiles extracted along the three orthogonal axes intersecting isocenter for dose distributions when treating 3 mm spherical target with a 2 x 5 mm^2^ aperture with 1.0 mm of linear motion along each axes. Black lines represent the case without motion, red lines represent the case with motion.

A visualization of the dosimetric impact of a linear motion (*L_1.0 mm_*) during treatment when irradiating a 3‐mm‐sized target with a 2 x 5 mm^2^ aperture is shown in Fig. 5. The volume within the target receiving the prescription dose is reduced by 26.3%, but when the same motion is implemented with a 4‐mm‐sized target, the volume receiving the prescription dose is only reduced by 11.0%. The hottest fraction of the target, represented by D_5_, is also reduced by motion, but by a smaller amount: 177.8% (no motion) vs 170.6% (with motion) for the 3‐mm target, and 133.8% (no motion) vs 139.6% (with motion) for the 4 mm target. When L*_1.0 mm_* motion is present, the dose wash area is reduced to 86.6, 84.5, and 84.3% in the axial, sagittal, and coronal planes along isocenter respectively when compared with a delivery without motion. An alternative visualization is shown in Fig. 6, where profiles are taken along the three orthogonal planes about isocenter when treating with a 2 x 5 mm^2^ aperture with *L_1.0 mm_* motion. It is evident that the dose intended for the TV can been pushed away and the shape of the dose distribution has changed.

**Fig. 7:**
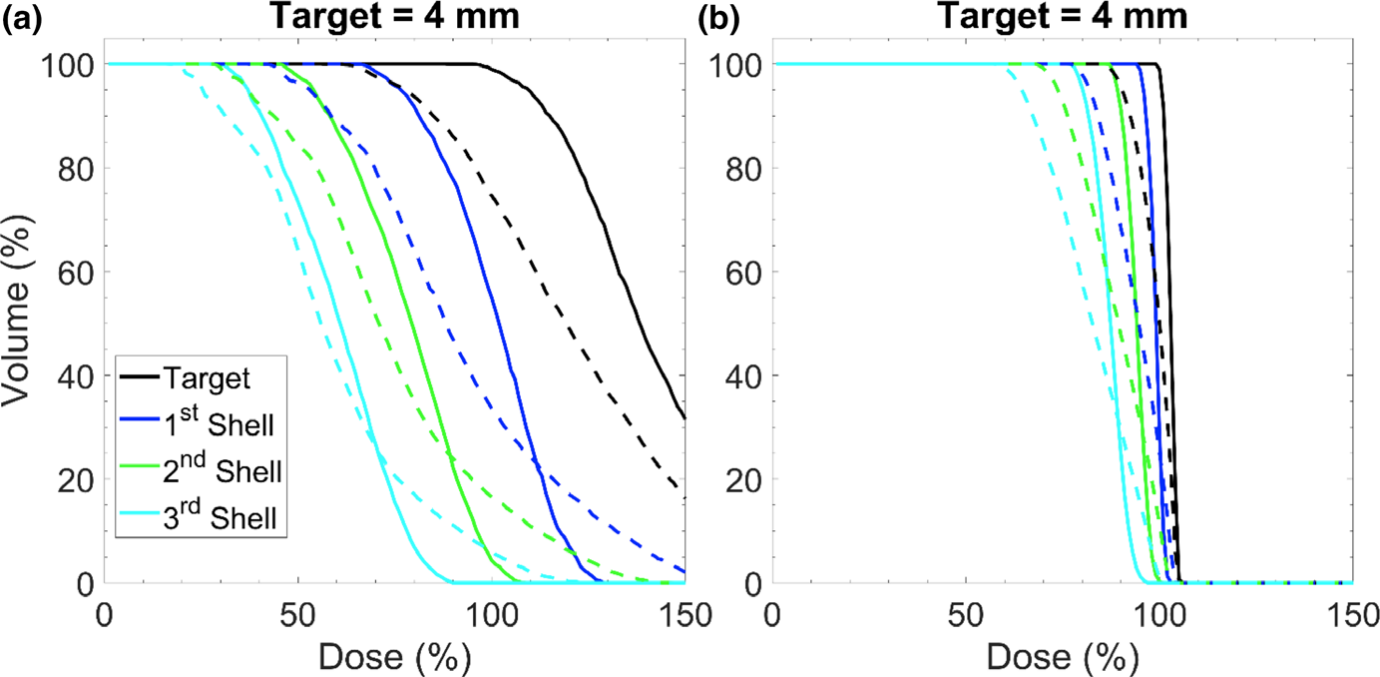
Dose volume histogram depicting impacts of motion for when the phantom has been moved linearly 1.5 mm along each dimension during treatment. (a) Represents an irradiation of a 4‐mm sized target with a 2 x 5 mm^2^ field size. (b) Represents an irradiation of a 4 mm sized target with a 6 x 10 mm^2^ field size. Each delivery was performed with the static collimator case. The solid lines represent delivery without motion and the dashed lines represent delivery with motion.

Analyzing the DVHs for two representative cases with the static collimator case; the dosimetric trade‐offs for different aperture sizes when intrafraction motion is present can be evaluated. In Fig. 7(a). and Fig. 7(b). these trade‐offs become apparent for an irradiation of a 4‐mm target irradiated with a 2 x 5 mm^2^, and 6 x 10 mm^2^‐sized field, respectively. As shown above in previous sections, irradiating with a smaller field has the potential to produce a sharper dose gradient as the surrounding concentric shells receive less dose. However, when intrafraction motion is present, small field sizes result in larger relative increases to the hotspots in the surrounding shells of tissue. In the example of Fig. 7, the increase in the D_5%_ for the smaller aperture (2 x 5 mm^2^) in the 1st, 2nd, and 3rd concentric shell was 39%, 24%, and 14% larger respectively when compared with the delivery using the larger aperture (6 x 10 mm^2^). While treating with a larger aperture minimizes the relative penalties of intrafraction motion, this comes at the expense of delivering a larger integral dose to the surrounding tissues.

**Fig. 8:**
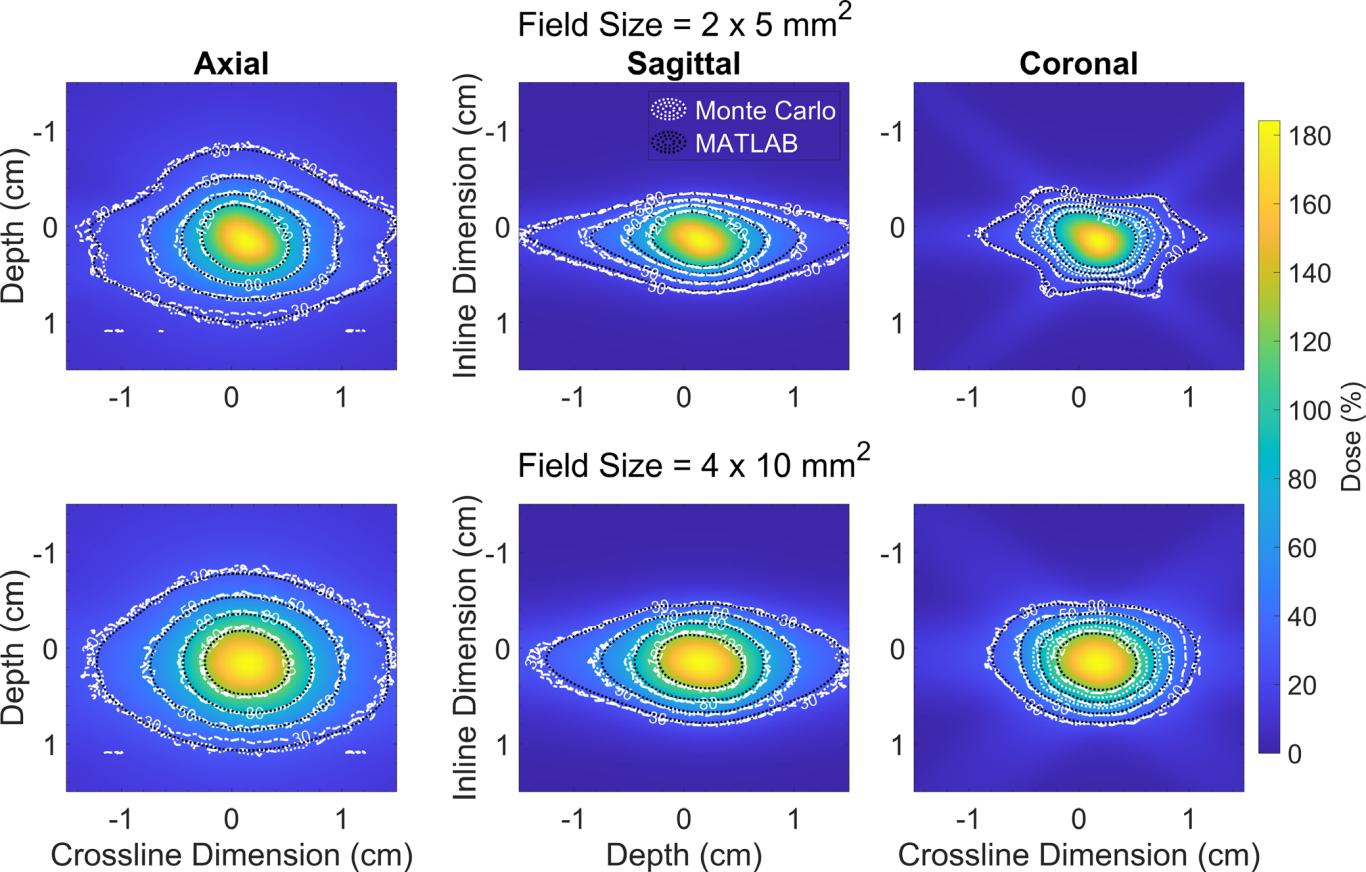
Isodose lines from dose distributions produced in Monte Carlo (white), and MATLAB (black) when simulation a sudden 2 mm shift ¼ of the way throughout treatment (S_1/4_). The isodose lines are overlayed on top of a dose wash produced using the MATLAB superposition methodology outlined in section 2.2. There are three write lines in the plot indicating the isodose lines for a central target (dotted), a target 2 cm off‐axis (dashed), and a target 3 cm off‐axis (dashed‐dotted). The lines all occupy effectively the same spaces, indicating that the off‐central location of targets is not playing a significant role in altering the dose distribution in these target locations.

The dosimetric impact of motion on targets that reside off‐axis are visualized in Fig. 8 where isodose lines for a full Monte Carlo treatment delivery with simulated motion (shown in white) are shown with isodose lines for the same treatment delivery using the proposed superposition methodology outlined in section 2.B (shown in black). The isodose lines for the different Monte Carlo deliveries appear virtually on top of each other for the treatment of a central target, a target 2 cm off‐axis, and a target 3 cm off‐axis. The isodose lines have been shifted for comparison with the dose distribution of the central target. In comparison to a delivery performed with the superposition methodology, D_5_ is −0.07%, 2.72%, and 5.16% different for the Monte Carlo delivery with the target centered, 2 cm, and 3 cm off‐axis, respectively. Similarly, the differences in V_100_ are less than 8 x 10^‐4^ cc for the three target locations when simulating a delivery with Monte Carlo.

**Fig. 9:**
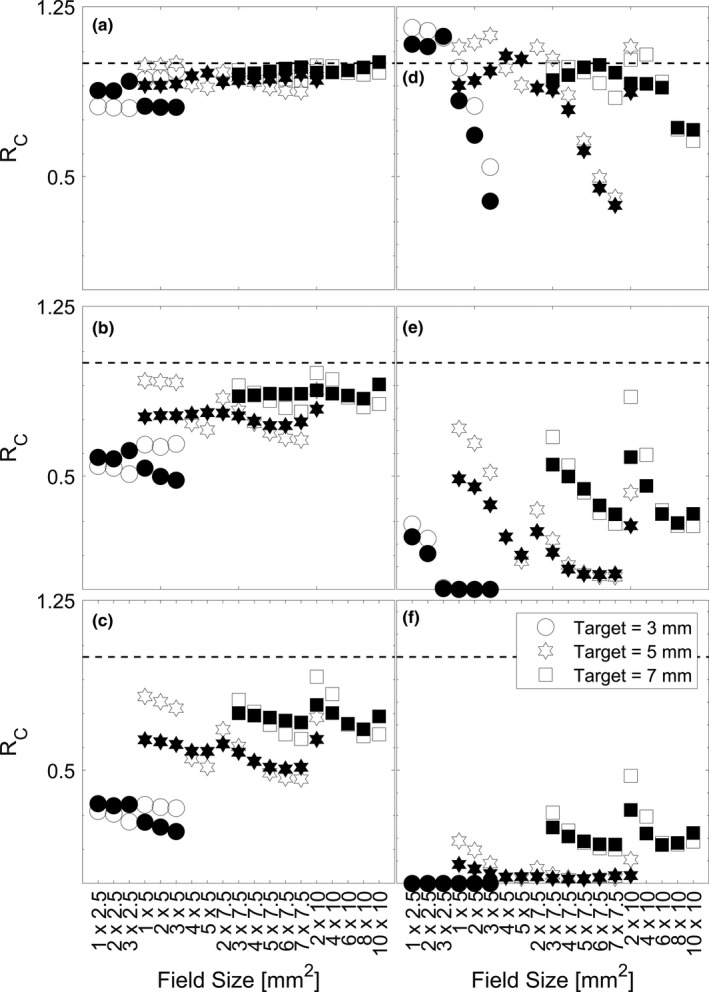
Ratio of the Paddick conformity index for deliveries with varying intrafraction motion. (A), (B), and (C) are plots for *L_0.5 mm_*_,_ *L_1.0 mm_*_,_ *L_1.5 mm_*_,_ which represents linear motion up to 0.5, 1.0, and 1.5 mm in each dimension respectively. (D), (E), and (F) are plots for *S _3/4_*_,_ *S _1/2,_* and *S_1/4_*_,_ which represents a linear motion of 2 mm in each dimension at ¾, ½, and ¼ of the way through treatment respectively. Open‐face symbols represent the static collimator case and closed‐face symbols represent the dynamic collimator case. The dashed line at unity represents the situation where a delivery with motion produces equal conformity to a delivery without motion.

In Fig. 9, the R_C_ is shown for all field sizes and each different type of motion trace. Predictably, the magnitude of conformity loss increases with increasing magnitude of linear drift. A similar trend is observed for the large shifts that occur at different time points, where earlier shifts producing larger losses of conformity. Interestingly, for the case of large shifts occurring a set time points in Figure D, E, and F, there is a trend of worsening conformity with increasing effective aperture area. The average R_C_ for the different cases of motion are summarized in Table 3.

**Table 3:** Average ratio of Paddick conformity index for the various cases of motion. The magnitudes and standard deviations are determined from averages across all apertures shown in Fig. 9

Type of Motion	Target Size (mm)		
	3	5	7
L*_0.5 mm_*	0.81 ± 0.07	0.93 ± 0.04	0.97 ± 0.02
*L_1.0 mm_*	0.49 ± 0.08	0.78 ± 0.09	0.89 ± 0.06
*L_1.5 mm_*	0.23 ± 0.07	0.61 ± 0.12	0.79 ± 0.10
*S*_3/4_	0.38 ± 0.45	0.83 ± 0.26	0.92 ± 0.09
*S*_1/2_	0.03 ± 0.08	0.30 ± 0.25	0.59 ± 0.20
*S*_1/4_	0.00 ± 0.00	0.09 ± 0.09	0.38 ± 0.20

**Fig. 10:**
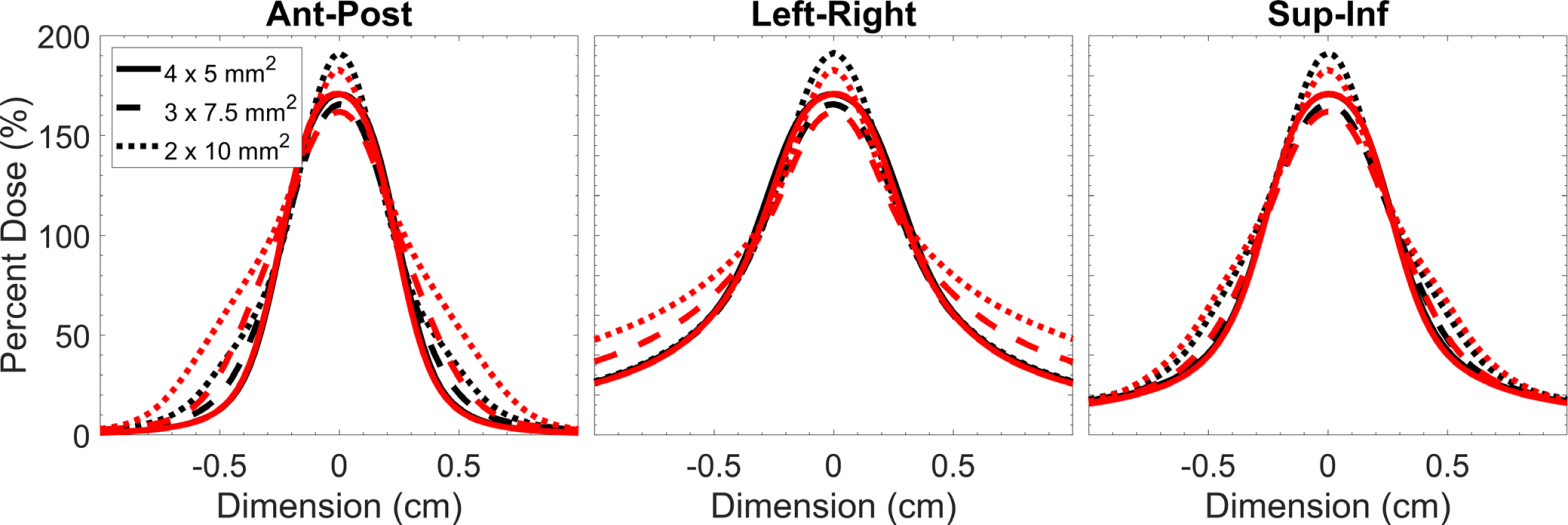
Dose profiles through the three orthogonal axis along isocenter for a delivery to a 5‐mm‐sized target with a 4 x 5 mm^2^, 3 x 7.5 mm^2^, and 2 x 10 mm^2^ field. Black lines represent the static collimator case and red lines represent the dynamic collimator case. Profiles were normalized to ensure that 99% of the target volume was covered by the prescription dose.

For any given target volume, the choice of an aperture, and collimator orientation technique will vary dose hot‐spots as well as the low–intermediate dose wash which influence the dosimetric conformity delivered to the target. This is visualized in Fig. 10 where dosimetric profiles along three orthogonal axes through isocenter have been extracted for the treatment of a 5‐mm target. The profiles are normalized to ensure that 99% of the target volume is covered by the prescription dose. In this figure, it is shown that while square‐like apertures (4 x 5 mm^2^) produce a steeper dose‐gradient outside of the target volume when compared with rectangular‐like aperture (3 x 7.5 mm^2^), they deliver a larger dosimetric hotspot (~5.2% larger), which could pose a larger detriment to surrounding sensitive structures; and the steepness of the dose gradient could lead to a larger decrement in target volume coverage when motion is present. The use of collimator rotation can be implemented to reduce the dosimetric hotspot (~8.4% as is depicted in the case of the 2 x 10 mm^2^ aperture) reducing the dosimetric risk to surrounding tissues when motion is present. While this also leads to a larger distribution of low–intermediate dose to surrounding tissues, this could minimize the decrements to conformity when motion is present for specific cases. For example, as is shown in Fig. 9(a)–9(c) when treating a 3‐mm‐sized target with a 1 x 2.5 mm^2^, 2 x 2.5 mm^2^, or 3 x 2.5 mm^2^ field when linear motion is present, using a dynamic collimator produces a 12% ± 3% higher R_C_. Seen across Fig. 9(a)–9(c) is the trend of a higher R_c_ with more rectangular apertures, as well as some values of R_c_ greater than unity. This effect is due to the relative shrinking of the target volume coverage in the case of motion when compared to the shrinking of prescription isodose volume in the case of motion. For example, when treating a 3‐mm sized target with as 1 x 2.5 mm^2^ field, the PIV_m_ is 63.4% of the PIV_no_, whereas the TV_m_ is 73.2% of the TV_no_, making the denominator (in the numerator of the equation in section 2.D) smaller, and the resulting quotient greater than unity.

## 4 Discussion

For the majority of the analysis considered in this work, many of the pairings of aperture size for a given target volume would be clinically impractical. The purpose of performing the analysis was to demonstrate the benefits and compromises one must make when considering TV coverage, hot spots, and magnitude of dose received to abutting tissues, and dose‐gradients. In the past decade, irradiation of lesions <1 cc. using VMAT, or dynamic conformal arc therapy have appeared for Brain Metastases^26^, ^27^ and Trigeminal Neuralgia.^24^ Popple *et al*. were the first to implement the use of a virtual cone with an arc‐based delivery for treating small targets such as trigeminal neuralgia with arc‐based and static port deliveries, respectively.^7^ That work determined that the target volume coverage by the 50% isodose line was 0.054–0.087 cc for a virtual cone shaped by the two central leaves of the MLC and a 1.6–2.6 mm gap. The data in this investigation agrees well as target volume coverage by the 50% isodose line is 0.051–0.093 cc for a virtual cone shaped by the two central leaves of the MLC and a 1–3 mm gap. For the range of all apertures tested in this investigation, a target volume coverage by the 50% isodose line is 0.022–1.359 cc.

It should be noted that when using an odd number of leaf pairs, the center of the treatment field is not located on the central axis and would require small couch motions to preserve target position relative to treatment aperture. Such motions have been demonstrated previously.^35^ Implications of mechanical imperfections in motion are not considered but have been considered in a previous investigation.^35^ A short‐coming of this investigation is the use of an 8.2 cm water sphere to represent a cranial phantom. This approximation was used to balance the computational requirements (time and memory) for conducting simulations with sufficient resolution. To test if this approximation had any impact on the dosimetric contribution of scatter, a single simulation with a 2 x 5 mm^2^ aperture was conducted with a cropped section of water sphere that measured 4 x 4 x 20 cm^3^, with the longest dimension along beam‐line, and a 0.4‐mm isotropic voxel size (time = 27.4 hrs). Using the same superposition methodology outlined above, the width of the dosimetric profiles defined by the 50% isodose line is 0.30 mm larger along ant‐post, 0.45 mm larger along sup‐inf, and 0.35 mm larger along left‐right when comparing the simulation of cranial phantom to the water sphere phantom.

Another limitation is the exploration of the dosimetric solution space when simulating intrafraction motion. There are an infinite number of choices that could be made when simulating motion traces during delivery. The varying degree of motions presented in this work: ***L_0.5mm_*** to ***L_1.5 mm_*** as well as ***S***_1/4_ to ***S***_3/4_ should provide clinicians with a meaningful way to consider the impact of motion in the context of their own clinic’s immobilization approaches. The motion traces used in this work were restricted to approaches utilized by other investigators to model the impact of motion on dose metrics,^21^, ^22^, ^23^, ^25^ and furthermore, to utilized average trends of motion that have been observed in the literature for cranial SRS.^14^, ^15^, ^16^, ^17^, ^18^, ^19^, ^20^

Neither of the collimation methods (static or dynamic) demonstrated a consistent dosimetric benefit; albeit, the 10% isodose line appeared to be most impacted by the dynamic collimator. The ranges for V_70_, V_50_, V_30_, and V_10_ are −0.043 to 0.060, −0.033 to 0.077, −0.078 to 0.156, and −0.789 to 0.579 cc, respectively. The dynamic collimator delivery led to a reduction in the in the high dose (V_70_), and low dose (V_10_) wash for the majority of cases, with the largest reductions occurring when the target size is ~60 to ~80% of the longest field size dimension and the length to width ratio of the field is ~1.25 to ~1.40 for spherical targets.

In this work, the impact of motion in the context of the volume receiving the prescription dose was highly variable across different aperture sizes, target sizes, and different magnitudes of motions. For a linear drift of 1.5 mm in each dimension (2.60 mm 3D‐shift vector), the ratio of the dose covering 95% of the volume in the case of motion to the case of no‐motion ranged from 53.55 to 98.23% of the no‐motion prescription dose. Without motion, the dose covering 95% of the volume is ≥100% of the prescription dose. Larger target volumes ≥5 mm exhibited a difference of V_95_ between 14.56% and 100% across all movement traces and smaller targets sometimes had 0% of their volume covered by the prescription dose due to dose‐blurring from motion. The magnitude of these differences are largely in agreement with Roper *et al*. which saw D_95_ <60%, and V_95_ <40% when considering 2° rotations during the treatment of lesions far from isocenter when irradiating multiple metastases with a single isocenter; as rotations to points far off‐axis would result in large perceived 3D‐shifts with respect to isocenter (similar to some movement traces simulated in this work).^26^

As shown in Fig. 10, the use of a dynamic collimator could push intermediate doses into a larger volume. This idea in conjunction with the size of aperture chosen (which dictates the prescription dose criteria that covers the target volume) leads to some values (shaded symbols) being higher in Fig. 8 when compared with the static collimator (open symbols). While not all data is shown, the static collimator produces a higher R_c_ in 59.6, 50.0, 52.6% of cases for the 3, 5, and 7 mm target, respectively; the clinical significance of cases where the dynamic collimator produces plans with an R_c_ closer to unity is unknown. Different applications of the dynamic collimator could be used for dose‐sparing in specific scenarios where sensitive structures abut the target volume and maximum dose tolerances have been reached.

## 5 Conclusions

In this work, we have demonstrated the dosimetric trade‐offs between aperture size and target size when irradiating with virtual cones. Larger apertures (effective area) produce smaller hotspots (D_5_) at the expense of delivering larger absolute doses to surrounding tissues. We have also shown the dosimetric impact of intrafraction motion consistent with previously published data derived from thermoplastic mask immobilization systems. For a given target size, the relative dosimetric penalties of intrafraction motion are smaller for larger aperture. In a representative example, the D_5%_ for a larger aperture (6 x 10 mm^2^) in the 1st, 2nd, and 3rd concentric shell was 39%, 24%, and 14% smaller, respectively, when compared with the delivery using the smaller aperture (2 x 5 mm^2^). Rotating the collimator throughout delivery is beneficial in minimizing the volumes covered by the intermediate dose wash in the majority of cases (50% and 30% of the prescription dose), but the clinical significance of these findings are unknown. Apertures with a larger length to width ratio minimized the reduction in conformity when motion is present. The data from this work illustrates the growing urgency and necessity for sub‐mm positioning when treating smaller targets.

## 6 Data Statement

The data that support the findings of this study are available from the corresponding author upon reasonable request.

## Financial Disclosures

CC, DP, and AS have no financial disclosures.
